# The Effect of Repetitive Whole Body Cryotherapy Treatment on Adaptations to a Strength and Endurance Training Programme in Physically Active Males

**DOI:** 10.3389/fspor.2022.834386

**Published:** 2022-03-25

**Authors:** Adnan Haq, William J. Ribbans, Erich Hohenauer, Anthony W. Baross

**Affiliations:** ^1^Sports Studies, Moulton College, Moulton, United Kingdom; ^2^Sport and Exercise Science, University of Northampton Waterside, Northampton, United Kingdom; ^3^School of Health, Sport and Professional Practice, University of South Wales Sport Park, Pontypridd, United Kingdom; ^4^The County Clinic, Northampton, United Kingdom; ^5^Department of Business Economics, Health and Social Care, University of Applied Sciences and Arts of Southern Switzerland, Landquart, Switzerland

**Keywords:** cryostimulation, cold, sport, exercise, fitness, power

## Abstract

Despite its potential merit in sport and exercise recovery, the implications of repetitive Whole Body Cryotherapy (WBC) during training programmes require further review due to the possibility of repetitive cold interfering with long term adaptations. This study investigated the impact of two weekly 3 min WBC sessions (30 s at −60°C, 150 s at −120°C) on adaptations to a 6 week strength and endurance training programme. Sixteen male participants (mean ± SD age 33.4 ± 9.8 years, body mass 82.3 ± 9.8 kg) randomly allocated into WBC (*n* = 7) and non-cryotherapy control (CON, n=9) groups completed the programme consisting of two weekly strength and plyometric training sessions and two weekly 30 min runs (70% VO_2_ max). Participants were assessed for body fat, VO_2_ max, muscle torque, three repetition maximum barbell squat and countermovement jump height before and after the programme. Resistance and running intensities were progressed after 3 weeks. Participants in both groups significantly improved muscle torque (WBC: 277.1 ± 63.2 Nm vs. 318.1 ± 83.4 Nm, *p* < 0.01, d = 0.56; CON: 244.6 ± 50.6 Nm vs. 268.0 ± 71.8 Nm, *p* = 0.05, *d* = 0.38) and barbell squat (WBC: 86.4 ± 19.5 kg vs. 98.9 ± 15.2 kg, *p* = 0.03, *d* = 0.69; CON: 91.1 ± 28.7 kg vs. 106.1 ± 30.0 kg, p < 0.01, d=0.51) following the 6 week programme. For the CON group, there was also a significant reduction in body fat percentage (*p* = 0.01) and significant increase in jump height (*p* = 0.01). There was no significant increase in VO_2_ max for either group (both *p* > 0.2). There was no difference between WBC and CON for responses in muscle torque, 3RM barbell squat and body fat, however WBC participants did not increase their jump height (*p* = 0.23). Repetitive WBC does not appear to blunt adaptations to a concurrent training programme, although there may be an interference effect in the development of explosive power. Sports practitioners can cautiously apply repetitive WBC to support recovery post-exercise without undue concern on athletes' fitness gains or long term performance, particularly throughout training phases focused more on general strength development than explosive power.

## Introduction

Whole Body Cryotherapy (WBC) is a potentially useful, albeit expensive tool for post-exercise recovery, demonstrating a variety of effects such as reductions in pain, swelling and inflammation (Lombardi et al., 2017). Whilst the treatment may benefit short term recovery (Hausswirth et al., 2011; Haq et al., 2021), athletes are concerned primarily with strategies to enhance longer term responses throughout a training cycle. One area of controversy is whether WBC might hinder adaptive responses to training.

Despite the demonstrated anti-inflammatory potential of WBC (Ziemann et al., 2012; Ferreira-Junior et al., 2014), the long term consequences of mitigating inflammation could be detrimental due to continual dampening of the adaptive responses. It is acknowledged that inflammation post-exercise is a means through which muscles can repair and regenerate (Fatourous and Jamurtas, 2016), thereby facilitating training adaptations.

Only four studies thus far have examined the potential impact of regular WBC treatment on adaptations to training. Broatch et al. (2019) discovered that three weekly WBC treatments did not influence adaptations to a 4 week cycling programme involving high intensity interval sessions. Growth factor benefits have been reported in volleyball and judo athletes following a 2 week period incorporating repeated WBC (10 total) and sports specific exercises (Jaworska et al., 2018, 2021). The same research group recently revealed that repetitive WBC (three times a week for 4 weeks) could support strength gains *via* reduced myostatin levels, an established negative mediator of muscle adaptations (Jaworska et al., 2020). These studies would therefore indicate no negative consequences of repetitive WBC application on adaptive responses.

On the contrary, numerous cold water immersion (CWI) studies indicate that repetitive cryotherapy can blunt training adaptations, particularly with regards to muscle strength and hypertrophy (Yamane et al., 2006; Roberts et al., 2015; Fyfe et al., 2019). Potential associated mechanisms include blunted arterial diameter gains and expression of anabolic signals (Yamane et al., 2006), attenuation of muscle fiber size increases as well as increased protein degradation markers (Fyfe et al., 2019) and blunted increases in testosterone (Earp et al., 2019). In contrast, other studies have not found such effects on endurance adaptations (Halson et al., 2014; Broatch et al., 2017). CWI has been demonstrated to augment endurance adaptations due to increased expression of PGC-1α, an established molecular marker in the activation of mitochondrial biogenesis (Ihsan et al., 2014).

Based on the findings of the aforementioned studies, it is plausible that repetitive cryotherapy treatments hinder resistance training adaptations more than endurance adaptations, a view supported by recent reviews (Bouzigon et al., 2021; Ihsan et al., 2021; Petersen and Fyfe, 2021). Physiological characteristics of resistance adaptations include increased motor unit recruitment, myofibril cross sectional area and increased number of sarcomeres (Schoenfeld, 2010; Del Vecchio et al., 2019). Whereas, endurance training is typically associated with enhanced responsiveness of blood vessels and muscle capillary density, increased nitric oxide levels, arteriogenesis, and mitochondrial biogenesis (Green et al., 2012; Hawley et al., 2018). Since it is established that the molecular pathways for each type of adaptation are distinct (Coffey and Hawley, 2017), impeding one type of pathway due to repetitive cooling does not necessarily imply a negative impact on the other pathway.

Additionally, the majority of the WBC and CWI studies highlighted previously only investigated training of a single type—i.e., interval or resistance training. Programmes incorporating a combination of training methods are arguably more relevant for overall sports practice with a focus on a variety of fitness attributes, including speed, power, strength, agility and endurance (Stolen et al., 2005; Wong et al., 2010). Research examining the impact of repetitive WBC treatment in conjunction with concurrent training programmes remains scarce. Whilst the WBC studies by Jaworska et al. (2018, 2021) utilized a combination of training methods, a 2 week training period is typically not long enough to induce sufficient adaptations (Kraemer et al., 1998). As such, it does not reflect a characteristic programme in populations aiming to significantly enhance strength, endurance and/or other key fitness attributes. Furthermore, mesocycles targeting specific fitness/performance markers in sport usually last at least 5 weeks (Marrier et al., 2018).

Certainly any negative impact of repetitive WBC on adaptive responses to training could outweigh the potential short term benefits, becoming counter-productive in the long run. This potential dilemma should be taken into consideration within the sports science community.

Due to the limited work and contrasting findings in this area, the overall impact of repetitive WBC treatment on chronic training adaptations remains unclear. Therefore, this study will aim to investigate the potential impact of repetitive WBC treatments on adaptations to a concurrent progressive 6 week training programme involving endurance, strength, and power training. Elucidating this effect should enable scientists and sports practitioners to further evaluate the overall merit of WBC treatment for performance gains, as well as having implications for cryotherapy usage in relation to the periodization of training schedules.

## Materials and Methods

### Participants

Twenty male volunteers were initially recruited for the study, with 10 randomly assigned as cryotherapy (WBC) and 10 assigned as non-cryotherapy control (CON). Three of the cryotherapy participants withdrew due to injury during their programme. One of the control participants withdrew due to testing positive for COVID-19 and developing prolonged symptoms. Thus, 16 participants completed the study (mean ± SD age 33.4 ± 9.8 years, height 1.79 ± 0.05 m, body mass 82.3 ± 9.8 kg). All participants were of a suitable fitness level for the demands of the study, partaking in physical activity at least twice weekly. Sample characteristics for each group are summarized in Table 1. Prior to assessment, all participants had their blood pressure assessed and signed an informed consent form. Ethical approval was obtained from the University of Northampton Graduate School Research Ethics Committee and the study was conducted according to guidelines of the Declaration of Helsinki.

**Table 1:** Summary of characteristics for whole body cryotherapy (WBC) and control (CON) participants in the training study.

	**WBC**	**CON**	**OVERALL**	**T-Test between groups**
	**(*n* = 7)**	**(*n* = 9)**	**(*n* = 16)**	
Age (y)	38.0 ± 10.0	29.8 ± 8.5	33.4 ± 9.8	*p* = 0.10
Height (m)	1.80 ± 0.04	1.79 ± 0.06	1.79 ± 0.05	*p* = 0.64
Body mass (kg)	80.7 ± 7.7	83.6 ± 11.5	82.3 ± 9.8	*p* = 0.58
Body mass index (kg/m^2^)	24.9 ± 2.2	26.1 ± 3.5	25.6 ± 3.0	*p* = 0.42
Body fat %	20.4 ± 5.5	19.4 ± 5.3	19.8 ± 5.3	*p* = 0.74
Absolute VO_2_ max (l/min)	3.75 ± 0.38	3.59 ± 0.53	3.66 ± 0.46	*p* = 0.53
Relative VO_2_ max (ml/min/kg)	46.7 ± 5.4	43.2 ± 5.6	44.8 ± 5.6	*p* = 0.23

*Data presented as mean ± SD*.

### Study Design

**Figure 1:**
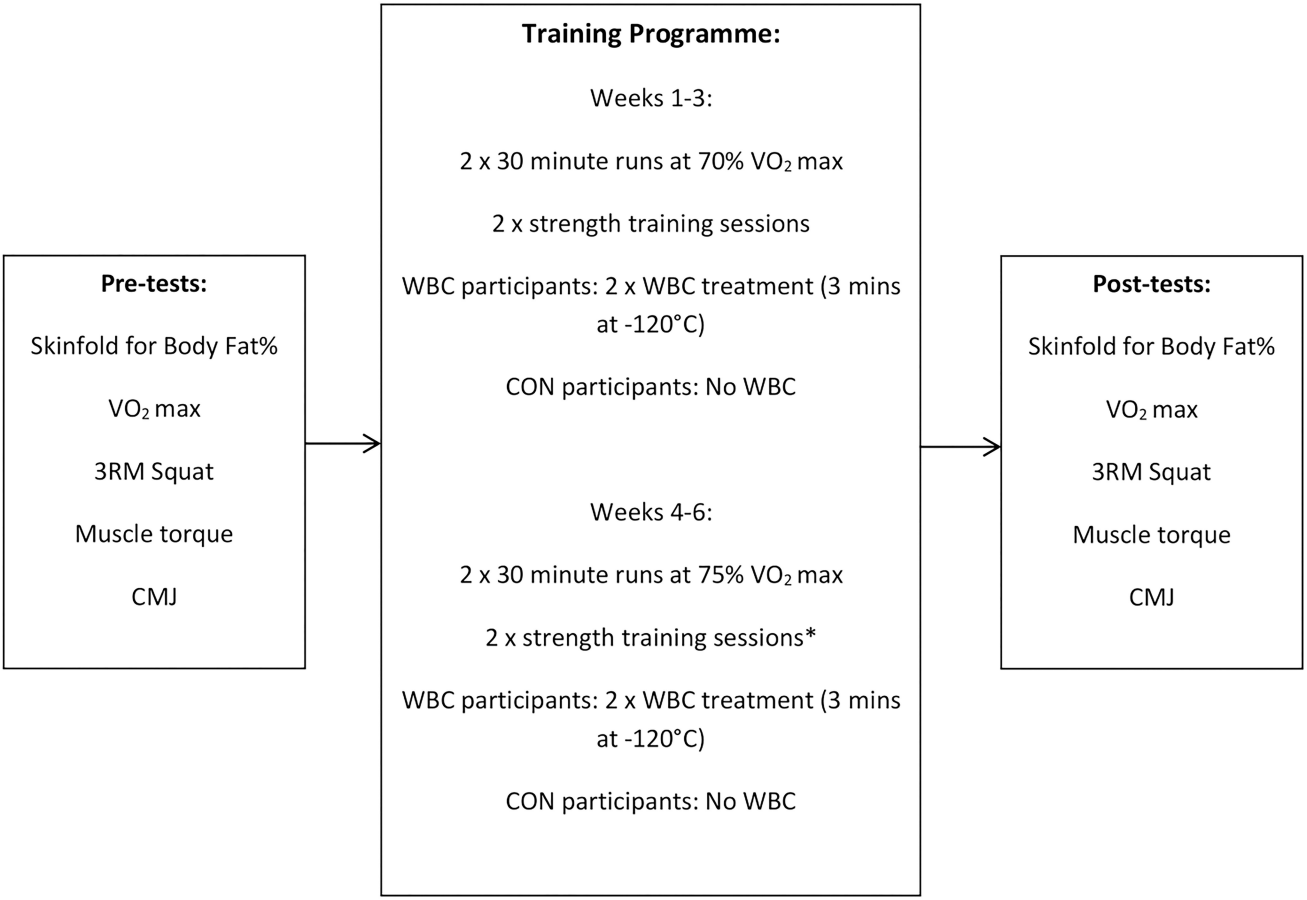
Summary of design for training study with 4 sessions performed weekly. WBC, whole body cryotherapy; CON, control; 3RM, 3 repetition maximum; CMJ, countermovement jump test. *Weight for barbell squat progressed from 70% 1RM to 75% 1RM after 3 weeks.

Participants performed a 6 week exercise training programme incorporating a mixture of endurance and strength training, with intensity increasing after 3 weeks. An independent groups design was employed where WBC participants performed their programmes in conjunction with two weekly WBC treatments. The CON group underwent the same training programme without cryotherapy intervention. To assess the impact of the training, pre and post tests were conducted on performance (VO_2_ max, muscle torque of the right quadriceps, three repetition maximum barbell squat, and countermovement jump height) and anthropometric characteristics (body fat). The study design is summarized in Figure 1.

### Experimental Procedures—Pre- and Post-test

All participants were asked to refrain from alcohol and strenuous exercise for 24 and 48 h, respectively prior to testing. Upon arriving for the first test, participants' blood pressure was assessed in a seated position. Anthropometric characteristics were assessed, including height and body mass. Body fat content was assessed by skinfold calipers (Harpenden Indicators, UK) using the methods described previously (Haq et al., 2021). Participants were familiarized to a muscle torque assessment using a Biodex dynamometer, which involved two submaximal isometric contractions (60 and 80% effort), followed by a singular maximal contraction.

Maximal aerobic capacity (VO_2_ max) was measured using an online breath by breath analyser (Cortex Metalyser, Germany), following an incremental treadmill (Cosmos, Germany) protocol until the participant reached volitional exhaustion, as described previously (Haq et al., 2021). The absolute and relative VO_2_ max values were reported and 70 and 75% of the absolute VO_2_ max were calculated.

Once fully recovered after the VO_2_ max test, participants underwent a barbell squat test where the maximum weight lifted for three repetitions was determined (Haff and Triplett, 2016). Participants performed 6–10 warm-up repetitions with a 20 kg Olympic barbell, ensuring appropriate technique (Myer et al., 2014). The weight was gradually increased by the addition of weight plates (Eleiko, Sweden) until participants attained the maximum weight they could complete the movement with correct form for three repetitions. Participants were given at least 2 min recoveries between each attempt. Participants were required to descend until their quadriceps were parallel to the ground and to fully extend at the knees on the upward phase. Proper activation of the gluteal muscles was also encouraged to ensure movement efficiency and reduced risk of muscle imbalances and injuries (Myer et al., 2014).

Unilateral isometric maximal torque of the right quadriceps was assessed by an isokinetic dynamometer (Biodex Medical Systems 3, New York) calibrated prior to the study. The dynamometer was fitted with a lever arm attachment locked in at 90° leg extension. Participants sat on the chair with 90° hip flexion using the setup described previously (Haq et al., 2021). Participants performed two warm up contractions at 60 and 80% effort, respectively, by exerting force against the pad. Following 2 min rest, they performed three maximal contractions (with 2 min recoveries) with verbal encouragement given throughout (Baross et al., 2013). All contractions were 5 s in duration. The peak torque (Nm) was determined as the maximum of the three contractions. A pilot study conducted in the laboratory revealed a day to day variance of 5.3% within individuals.

The final exercise test was a maximal countermovement jump (CMJ) for the assessment of explosive power. Participants were required to vertically jump on a mat (Perform Better, UK), aiming for maximum height five times whilst keeping their legs extended and hands on hips prior to landing. Participants were asked to land in the same position each jump (Markovic et al., 2004). The flight times (ms) and heights (mm) were measured for each jump. Flight time was measured as the interval between lift-off and landing of the feet and automatically converted into jump height. The means from the highest three jumps were recorded as the individuals pre-test score (Young et al., 1995). The CMJ has been demonstrated to be a reliable test for jump power, effectively activating the stretch-shortening cycle and with low reported within-subject variance (2.8%) and high correlation coefficient (0.98; Markovic et al., 2004).

Participants returned to the laboratory 3–7 days following their final training session to have the same six tests re-assessed in the same order to determine their post-test score. Caffeine, alcohol and strenuous activity were avoided as before and the post-test was conducted at a similar time of day as the pre-test.

### Training Programme and Exercises

A 6 week training duration was considered an appropriate compromise between attaining significant adaptations (Kraemer et al., 1998; Marrier et al., 2018) and timing/logistical constraints (e.g., use of the cryotherapy chamber). Participants commenced their training programme between 3 and 7 days following the pre-test. The programme consisted of four sessions per week: two 30 min treadmill runs at 1% incline and initial intensity equivalent to 70% VO_2_ max, as well as two strength and plyometric training sessions with 2 min recoveries between sets. There were five exercises: barbell squats (4 × 6 repetitions at 70% estimated one repetition maximum); dumbbell lunges (3 × 8 on each leg); nordic leg curls (2 × 8); depth jumps (3 × 8 from a box height of 30 cm) and split lunge jumps (3 × 8 on each leg with hands on hips). Participants were familiarized with all exercises beforehand.

The four sessions were performed on separate days of the week in the order of run>weights>run>weights. Since many of the participants were previously untrained, a frequency of four total sessions was considered an appropriate balance between attaining adaptations and preventing overtraining and/or injury. The WBC participants received cryotherapy treatment within 30 min following completion of their first and final training sessions each week.

After 3 weeks, the treadmill speed progressed to an intensity corresponding to 75% of initial VO_2_ max and the barbell squat weight increased to 75% of estimated 1RM to ensure appropriate progressive overload. Participants were instructed to continue with their usual diets and activity levels outside of the prescribed sessions.

### Verification of Training Running Speeds

Following completion of the pre-tests and sufficient recovery, participants were linked to the gas analyser to determine their prescribed treadmill running speeds. For the first 3 weeks of the programme, participants were required to run at a speed eliciting an intensity equivalent to 70% of absolute VO_2_ max. The intensity progressed to 75% VO_2_ max for the second 3 weeks. Participants ran on the treadmill (1% incline) at a steady speed with VO_2_ values continuously monitored. The speed was slightly adjusted until a VO_2_ value equivalent to 70% VO_2_ max was attained on a consistent basis. Expired gas data was averaged over a period of at least a minute of running at a constant speed during this process. Following establishment of the speed at 70%, the speed was progressed slightly and the same process was applied for determination of the speed at 75% VO_2_ max.

### Whole Body Cryotherapy Treatment

Cryotherapy treatments were undertaken in a two-stage cryogenic chamber (JUKA, Poland). The source of cold was liquid cryogenic gas originating from external pressure vessels. Participants were screened for contraindications following the completion of a health questionnaire, including hypertension, other cardiovascular diseases, open wounds, cold intolerance, and neural/mental disorders. Before entering the chamber, participants wore a head band, face mask, gloves, socks, elbow and knee bands, and clogs. Participants entered the cryotherapy chamber, initially exposed to a vestibule chamber at −60°C for 30 s, followed by the main chamber at −120°C for 150 s (Haq et al., 2021). On completion, the exit door for the main chamber opened and the participant exited. Thereafter, participants were advised to stay mobile before changing in usual clothing.

### Statistical Analysis

All data was analyzed using SPSS Version 26. Data for all variables was assessed for normal distribution by the Shapiro-Wilk test. There was no significant deviation from normality in any of the variables except for 3RM squat, where a log transformation was applied. A two way repeated measures analysis of variance was used to assess the interaction effect between treatment group (WBC vs. CON) and time (pre vs. post) for all five outcome variables. Paired *t*-tests were applied to examine pre-post differences within the WBC and CON groups. Effect sizes (Cohen's d) were calculated and defined as follows: small-−0.2; medium-−0.5; large-−0.8 (Cohen, 1992). For each dependent variable, 95% confidence intervals (CI) were also calculated. Significance levels were set at 0.05.

## Results

### Anthropometry

**Figure 2:**
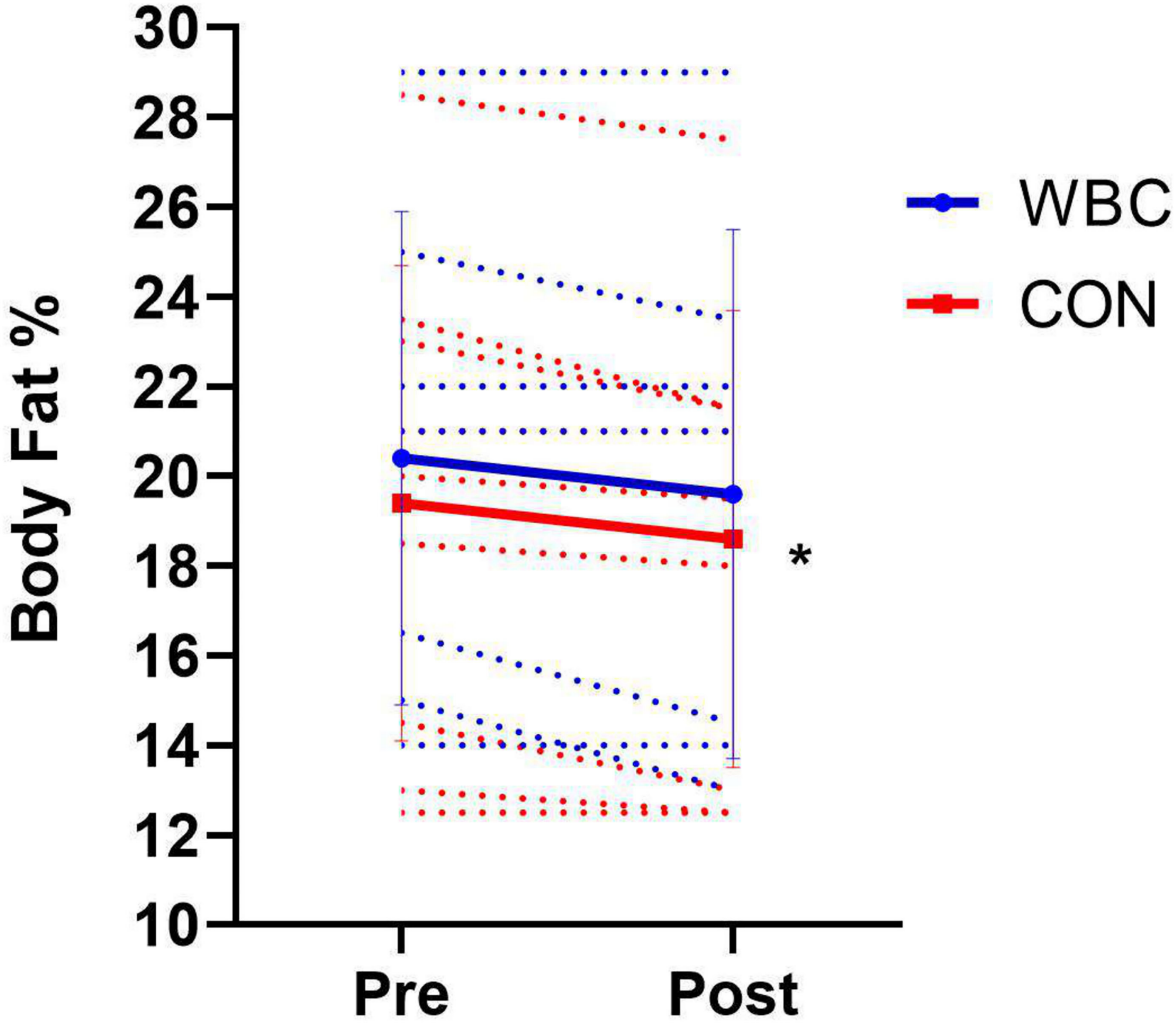
Body fat percentages before and after the 6 week training programme for WBC (*n* = 7) and CON (*n* = 9) groups. *p <0.05 for body fat decrease in CON. Solid lines present means ± standard deviations. Dotted lines present individual participant responses. *N* = 16.

Body fat percentage significantly decreased following the 6 week training programme for the CON group (19.4 ± 5.3%, 95% CI [15.9, 22.9] vs. 18.6 ± 5.1%, 95% CI [15.2, 21.9], *p* = 0.01, *d* = 0.16) whilst the decrease approached significance for the WBC group (20.4 ± 5.5%, 95% CI [16.3, 24.5] vs. 19.6 ± 5.9%, 95% CI [15.2, 24.0], *p* = 0.08, *d* = 0.14, Figure 2). There was no difference between groups over time (interaction effect, *p* = 0.90, *d* = 0.05). There was no difference in body mass before and after the training for either group (WBC, 80.7 ± 7.7 vs. 80.7 ± 9.3 kg, *p* = 1.0; CON, 83.6 ± 11.5 vs. 84.2 ± 10.6, *p* = 0.24).

### Relative VO_2_ Max

**Figure 3:**
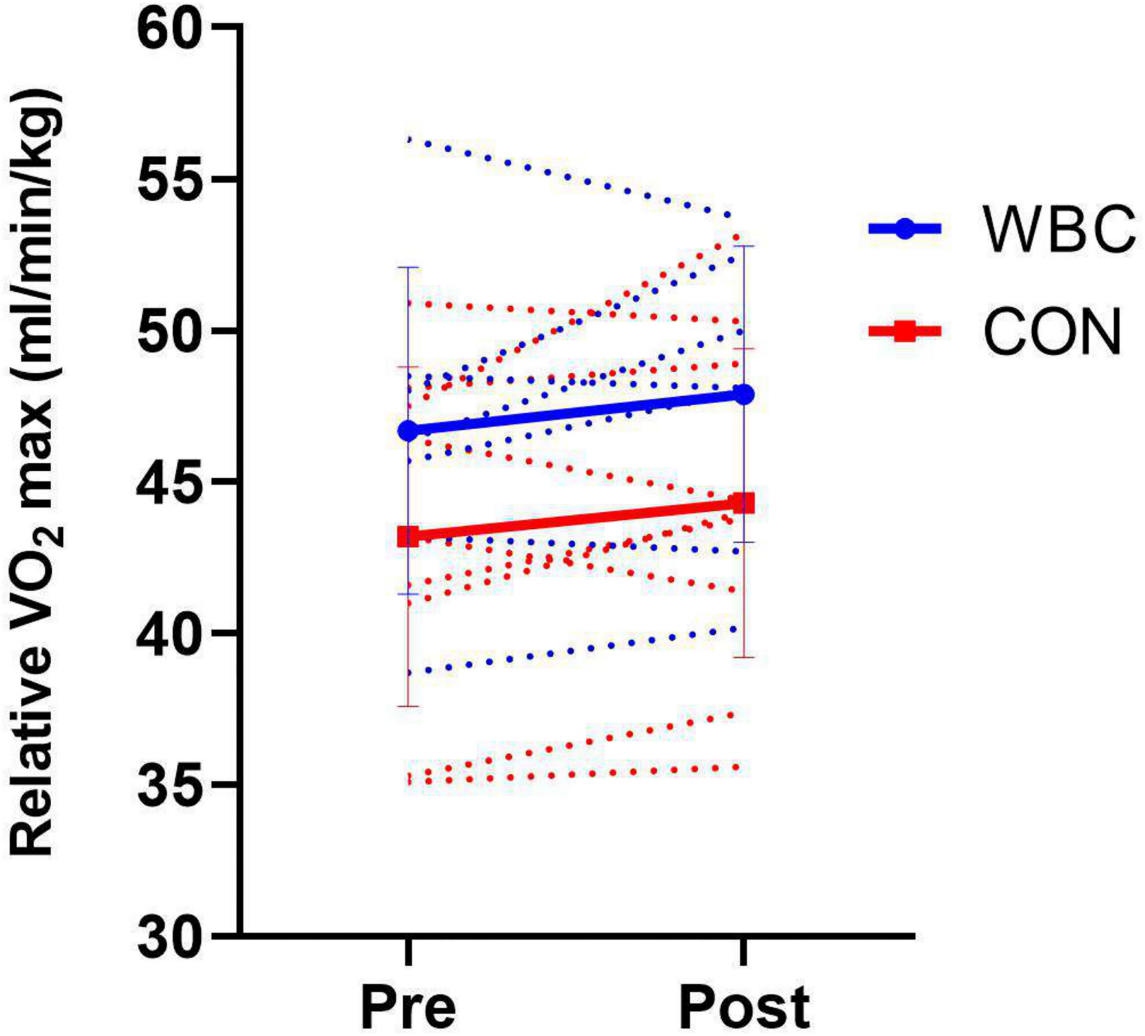
Relative VO_2_ max for WBC (*n* = 7) and CON (*n* = 9) groups before and after 6 week training programme. Solid lines present means ± standard deviations. Dotted lines present individual participant responses. *N* = 16.

There was no significant increase in relative VO_2_ max following the training programme for either WBC (46.7 ± 5.4 ml/min/kg, 95% CI [42.7, 50.7] vs. 47.9 ± 4.9 ml/min/kg, 95% CI [44.2, 51.5], *p* = 0.25, *d* = 0.23) or CON (43.2 ± 5.6 ml/min/kg, 95% CI [39.6, 46.9] vs. 44.3 ± 5.1 ml/min/kg, 95% CI [41.0, 47.6], *p* = 0.24, *d* = 0.20, Figure 3). There was no difference between groups over time (interaction effect, *p* = 0.90, *d* = 0.04).

### Muscle Torque

**Figure 4:**
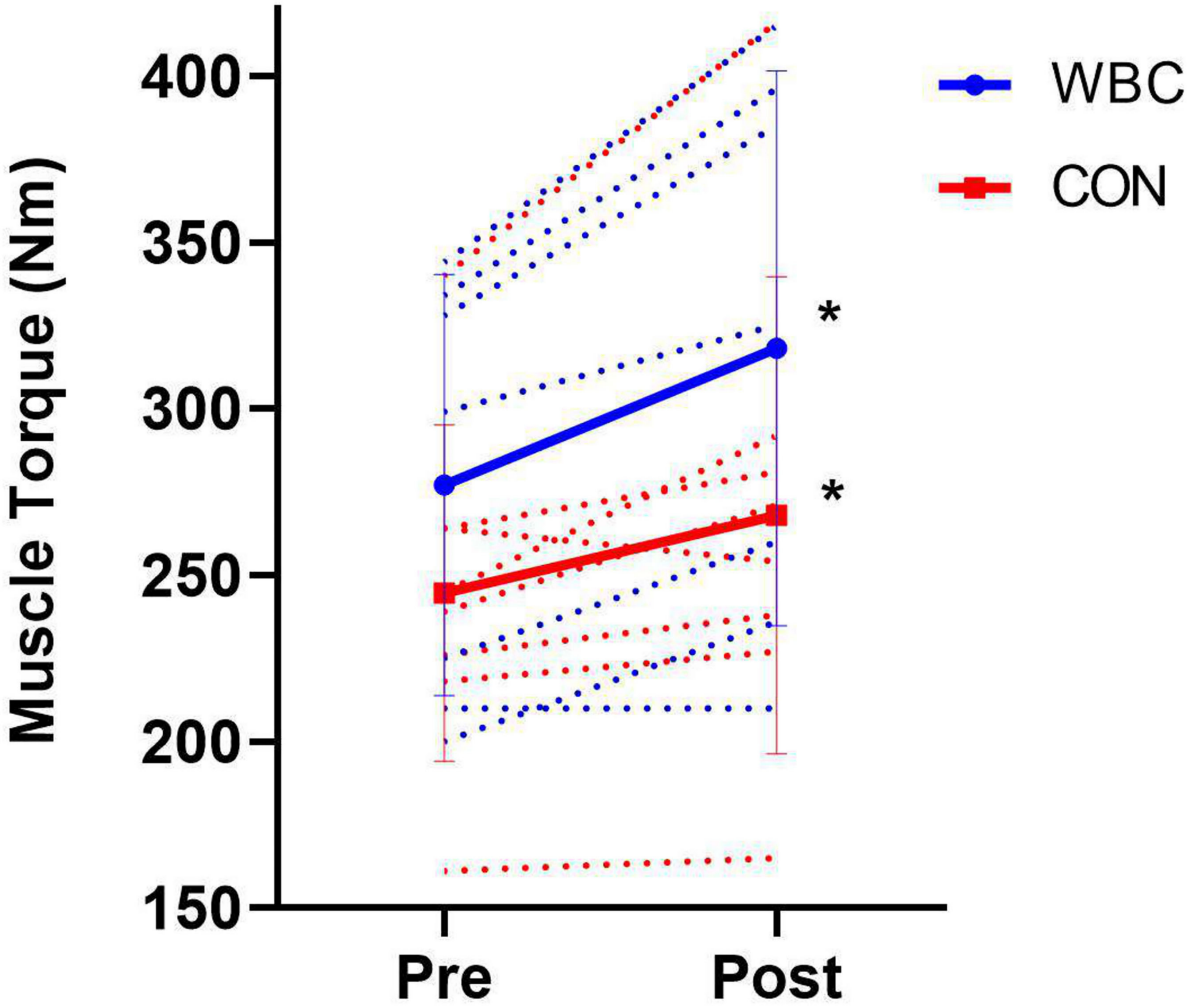
Leg muscle torques for WBC (*n* = 7) and CON (*n* = 9) before and after 6 week training programme. *p ≤ 0.05 for increase in both groups. Solid lines present means ± standard deviations. Dotted lines present individual participant responses. *N* = 16.

There was no significant difference in baseline muscle torques between groups (*p* = 0.29). Maximal isometric leg muscle torque significantly increased following the training in both WBC and CON groups (WBC, 277.1 ± 63.2 Nm, 95% CI [230.3, 323.9] vs. 318.1 ± 83.4 Nm, 95% CI [256.32, 380.0], *p* = 0.00, *d* = 0.56; CON, 244.6 ± 50.6 Nm, 95% CI [209.6, 279.7] vs. 268.0 ± 71.8 Nm, 95% CI [218.3, 317.7], *p* = 0.05, *d* = 0.38, Figure 4). There was no significant difference between groups over time (interaction effect, *p* = 0.21, *d* = 0.65).

### Barbell Squat

**Figure 5:**
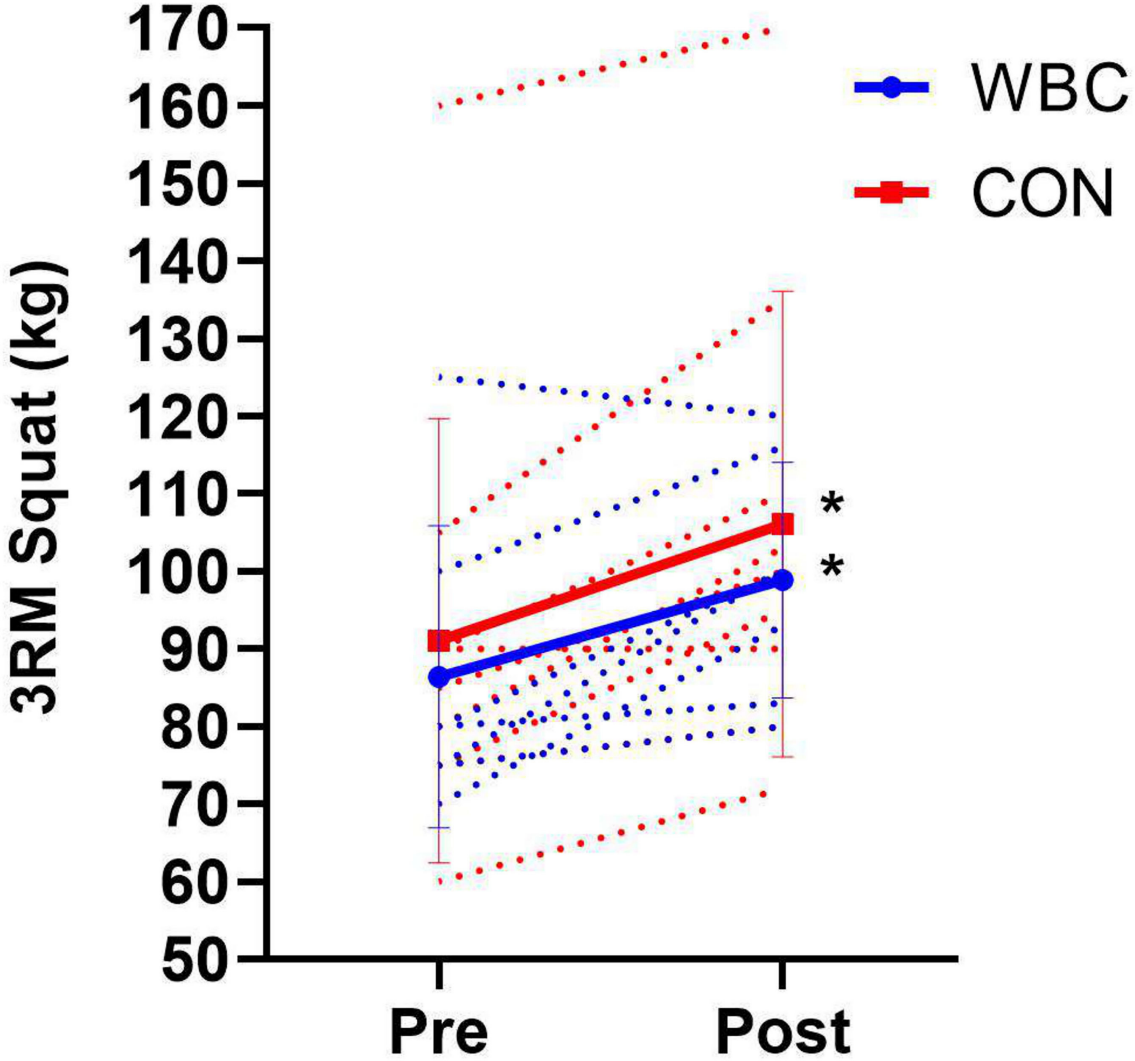
Barbell squat three repetition maximums for WBC (*n* = 7) and CON (*n* = 9) before and after 6 week training programme. *p ≤ 0.05 for increase in both groups. Solid lines present means ± standard deviations. Dotted lines present individual participant responses. *N* = 16.

There was no difference in baseline between groups (*p* = 0.78). Three repetition maximum squat significantly increased in both groups following the training (WBC, 86.4 ± 19.5 kg, 95% CI [72.0, 100.9] vs. 98.9 ± 15.2 kg, 95% CI [87.6, 110.1], *p* = 0.03, *d* = 0.69; CON, 91.1 ± 28.7 kg, 95% CI [72.4, 109.9] vs. 106.1 ± 30.0 kg, 95% CI [86.5, 125.7], *p* = 0.00, *d* = 0.51, Figure 5). There was no difference between groups over time (interaction effect, *p* = 0.80, *d* = 0.10).

### Countermovement Jump

**Figure 6:**
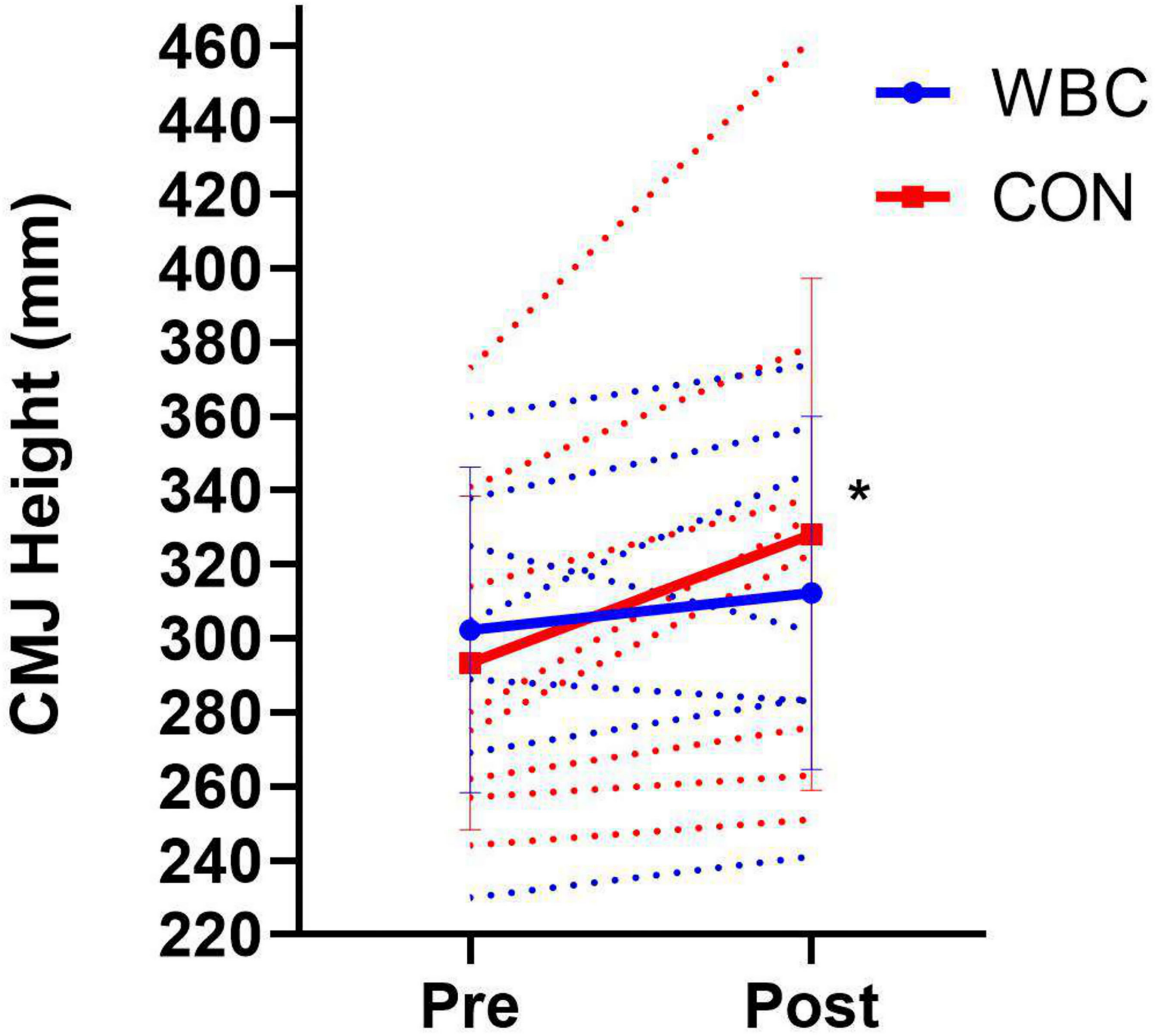
Countermovement jump height for WBC (*n* = 7) and CON (*n* = 9) before and after 6 week training programme. *p <0.05 for increase in CON group. Solid lines present means ± standard deviations. Dotted lines present individual participant responses. *N* = 16.

There was no difference in baseline jump height between groups (*p* = 0.70). Vertical jump height did not significantly increase in the WBC group following the training programme (302.3 ± 44.0 mm, 95% CI [269.7, 334.9] vs. 312.3 ± 47.8 mm, 95% CI [276.9, 347.7], *p* = 0.23, *d* = 0.22), whilst there was a significant increase for the CON group (293.3 ± 45.2 mm, 95% CI [261.9, 324.6] vs. 328.1 ± 69.2 mm, 95% CI [280.1, 376.1], *p* = 0.01, *d* = 0.61, Figure 6). The overall interaction between group and time approached significance (*p* = 0.07, *d* = 0.96).

## Discussion

This is one of the first studies to investigate the impact of repetitive WBC treatment on adaptations to a strength and endurance training programme. The 6 week concurrent and progressive training programme was generally effective in improving lower body strength, jump power and body composition in both groups. However, there were no significant gains in aerobic endurance. Repetitive whole body cryotherapy did not appear to have a detrimental impact on such fitness attributes; however it might hinder improvements in countermovement jump power.

Prior studies on cold water immersion (CWI) treatments (Roberts et al., 2015; Fyfe et al., 2019) indicate that repetitive cryotherapy is unfavorable and potentially damaging to long term training adaptations. This could be due to attenuation of anabolic signaling and muscle protein synthesis (Petersen and Fyfe, 2021), as well as potential dampening of the inflammatory response which would otherwise be a necessary component of adaptive gains (White and Wells, 2013). This current study indicates that repetitive cryotherapy does not hinder such adaptations, particularly with regards to muscle strength. However, the lack of significant increase in countermovement jump height for the cryotherapy group indicates that repetitive WBC may hinder adaptations in explosive power which would be of concern to athletes where power is an important performance component.

Previous studies that have examined the influence of WBC treatments on training responses adopted training of a single type—interval (Broatch et al., 2019) or resistance (Jaworska et al., 2020) or were a much shorter duration of just 2 weeks (Jaworska et al., 2018, 2021). The inclusion of a mixture of endurance, strength and power training in this study is arguably more representative of sports training programmes. The 24 training sessions performed in this programme is also substantially more than the aforementioned studies. It is therefore likely that the overall higher load in this 6 week programme would induce more significant adaptations.

The training programme was effective in improving the participants' body compositions following the 6 week programme, as evidenced by the significant decreases in body fat percentages assessed *via* skinfold (Figure 2). Since there was no difference in body mass before and after the programme, it is highly probable that the participants increased their lean muscle mass. Increases in lean body mass are a common observation following exercise training programmes, particularly when the exercises are unaccustomed (Brook et al., 2015; Benito et al., 2020). Repetitive WBC treatments did not blunt this adaptive response (0.8% decrease in body fat for both WBC and CON groups). Therefore, sports competitors and practitioners do not need to be concerned about any potential detrimental impact of such treatments on body composition following a training programme.

The outcomes of this study would suggest that it is unlikely that WBC treatment can be used as an effective tool to reduce body fat and improve body composition in conjunction with an athletic training programme. However, results may differ with a higher frequency of weekly WBC treatments. The relationship between body composition and sports performance has already been well-documented (Collins et al., 2014; Esco et al., 2018). It is theorized that cold treatments could support changes in body composition *via* increased thermogenesis, activity of brown adipose tissue and subsequent caloric burn and fat loss (Cannon and Nedergaard, 2012). Pilch et al. (2020) recently demonstrated fat mass decreases following repetitive WBC, however no experimental study has showed promising results regarding repetitive WBC and body fat content in a sports or training context.

There were clear improvements in muscle strength following the training programme (Figures 4, 5), as assessed by both barbell squat performance and maximal isometric muscle torque. Numerous studies have documented the relationship between lower body strength and sports performance (Seitz et al., 2014) as well as the benefits of the barbell squat exercise in sports training programmes (Clark et al., 2012; Vecchio et al., 2018). The improvements in barbell squat three repetition maximum and muscle torque were comparable in both WBC and CON groups, indicating that repetitive cryotherapy treatment does not attenuate the adaptive gains of muscular strength following a strength training programme. This study did not assess protein synthesis rates or muscle fiber cross sectional areas. Yet, it is conceivable that due to the likely increases in lean body mass, the strength gains in both groups were the result of a combination of muscle hypertrophy, neurological adaptations and superior motor unit recruitment (Haff and Triplett, 2016; Del Vecchio et al., 2019). A cautionary note is that it would be difficult to conclude that repetitive WBC can augment strength adaptations compared to CON, despite a moderate effect of 0.65 for between group differences in muscle torque.

The outcome of the lack of difference in strength response between WBC and CON groups contradicts previous findings of repetitive CWI attenuating gains in muscular strength (Roberts et al., 2015). The fairly modest quantity of 12 cryotherapy treatments in total could be a factor in the lack of hindrance effect on adaptations. An alternative explanation is that the modality and mechanisms of the cold exposure in WBC is different to that of CWI (Bleakley et al., 2014). Cold water exerts a hydrostatic effect and has a higher thermal conductivity than cold air (White and Wells, 2013). The latter may therefore not hinder subsequent amino acid supply to muscles and anabolic cell signaling to the same extent. This potential explanation is supported by findings that CWI elicits more pronounced reductions in lower limb blood flow than WBC (Mawhinney et al., 2017). Further studies on repetitive WBC treatment in training programmes are necessary to form conclusions regarding the potential impact and mechanisms of repetitive cold exposures on attenuating gains in muscle strength/hypertrophy.

Whilst WBC has previously shown to benefit muscle power in the short term (Fonda and Sarabon, 2013) and could support post-activation potentiation following exercise (Partridge et al., 2019), the impact of repetitive WBC on long term muscle power development remains inconclusive. It was initially the authors' intention to include power specific exercises to take advantage of activating the stretch shortening cycle, which would more likely be representative of sports training programmes. For instance, the depth jump exercise has previously been demonstrated to be effective in eliciting power improvements (McClenton et al., 2008). Countermovement jump height significantly improved in the CON group (293.3 vs. 328.1 mm), indicating that the programme was suitable for training leg power. However, the cryotherapy group did not demonstrate such improvements (Figure 6). Combined with the large between group effect size of 0.96, this might indicate that repetitive WBC blunts the adaptive response to gains in power. The possible physiological mechanisms for this blunted response are not clear, particularly when the cryotherapy group significantly improved their muscle strength, which is an important component of power (Kraemer and Looney, 2012). Whilst the total force output was unaffected, the rate of force development could have been compromised by repetitive WBC applications. Possible mechanisms for this attenuation could be a reduction in motor unit activation and/or signaling frequency from the central nervous system. The cryotherapy participants did not appear to improve their stretch shortening cycle capabilities, potentially compromising the elastic properties of the muscle despite improvements in total force capacities (Kraemer and Looney, 2012). A recent CWI study has indicated negative effects of repetitive cold on power development based on lack of CMJ improvements (Fyfe et al., 2019). The possible mechanisms in which repetitive cold could compromise the rate of force development is an area that requires further research before conclusions can be drawn regarding the effects.

The main finding that repetitive WBC did not blunt strength adaptations would have positive implications for sports periodization and training programming, yet the possible negative impact on explosive power development remains inconclusive. It may consequently be preferable for athletes to undergo repetitive WBC treatments throughout training phases when there is higher priority on general strength development than explosive power. For instance, strength development would be prioritized for many sports during the general adaptation phase where there would be more focus on taking advantage of the general adaptation syndrome *via* supercompensation (Turner, 2011). Alternatively, repetitive WBC can be applied during competitive or tapering phases of athletic training programming, when athletes are more concerned with exercise recovery as opposed to training adaptations.

## Study Limitations

The relatively small sample size for the cryotherapy group should be taken into account before interpreting the results, particularly in concluding that repetitive cryotherapy blunts adaptations in muscular power. Whilst it is unlikely that a larger sample would substantially alter the outcomes regarding strength and VO_2_ max (due to similar responses to the CON group), it is conceivable that the outcome in jump height was impacted by this sub-optimal sample.

Another potential factor is that the lack of impact of repetitive cryotherapy treatments on adaptive responses to training may be due to the relatively low number of treatments. Higher frequencies of WBC treatments are more likely to significantly attenuate adaptive inflammatory responses (Zembron-Lacny et al., 2020). Logistical and timing constraints in this study meant that it was not feasible for each participant to perform more than two weekly cryotherapy treatments. Additionally, the economic viability of higher numbers of weekly WBC treatments requires further consideration.

Finally, whilst the variables were measured pre and post-training to evaluate the overall adaptive response, little insight is provided into how effective the WBC treatments were in promoting short term recovery following the exercise sessions. Previous work has demonstrated that WBC treatments can effectively mitigate muscle strength reductions post-muscle damaging exercise (Haq et al., 2021), however it is not clear if the WBC interventions in this programme were equally effective for this purpose. Due to logistical and timing constraints, it was not possible to assess other physiological or performance parameters throughout the training programme (e.g., blood markers, muscle strength reductions). Thus, the specific mechanisms of the WBC treatments in this study and how they could influence adaptive responses to the training remains open to interpretation.

## Conclusions

In conclusion, repetitive WBC treatment did not have a significant negative impact on adaptations to muscle strength or body composition following a concurrent 6 week training programme. WBC may therefore be a preferable modality to CWI for repetitive applications during training cycles. Sports practitioners can cautiously apply repetitive WBC to support recovery post-exercise without undue concern on athletes' fitness gains or long term performance, particularly throughout training phases focused more on general strength development than explosive power. However, the potential negative impact on muscular power remains inconclusive. Further research should focus more on clarifying the potential mechanisms by which repetitive WBC can affect physiological adaptations to sports training programmes, particularly with regards to muscle power. It would be beneficial for future studies to measure parameters such as electromyography and muscle hypertrophy markers (e.g., protein synthesis rates and muscle fiber cross sectional areas) in order to elucidate how repetitive WBC could mediate potential mechanisms of strength and power development.

## Data Availability Statement

The raw data supporting the conclusions of this article will be made available by the authors, without undue reservation.

## Ethics Statement

The studies involving human participants were reviewed and approved by University of Northampton Graduate School Research Ethics Committee. The patients/participants provided their written informed consent to participate in this study.

## Author Contributions

AH, WR, and AB contributed to the study conception and design. AH performed the material preparation, data collection, data analysis, and wrote the first draft of the manuscript. Supervision by WR and AB. AH, WR, EH, and AB reviewed and edited previous versions of the manuscript, read, and approved the final version of the manuscript.

## Funding

Open Access Publication fees to be funded by University of Applied Sciences and Arts of Southern Switzerland (SUPSI).

## Conflict of Interest

The authors declare that the research was conducted in the absence of any commercial or financial relationships that could be construed as a potential conflict of interest.

## Publisher's Note

All claims expressed in this article are solely those of the authors and do not necessarily represent those of their affiliated organizations, or those of the publisher, the editors and the reviewers. Any product that may be evaluated in this article, or claim that may be made by its manufacturer, is not guaranteed or endorsed by the publisher.

## References

[B1] Baross A. W. Wiles J. D. Swaine I. L. (2013). Double-leg isometric exercise training in older men. Open Access J. Sports Med. 4, 33–40. 10.2147/OAJSM.S39375 24379707 PMC3871900

[B2] Benito P. J. Cupeiro R. Ramos-Campo D. J. Alcaraz P. E. Rubio-Arias J. Á. (2020). A Systematic review with meta-analysis of the effect of resistance training on whole-body muscle growth in healthy adult males. Int. J. Environ. Res. Pub. Health 17:1285. 10.3390/ijerph17041285 32079265 PMC7068252

[B3] Bleakley C. M. Bieuzen F. Davison G. W. Costello J. T. (2014). Whole-body cryotherapy: empirical evidence and theoretical perspectives. Open Access J. Sports Med. 10, 25–36. 10.2147/OAJSM.S41655 24648779 PMC3956737

[B4] Bouzigon R. Dupuy O. Tiemessen I. De Nardi M. Bernard J. P. Mihailovic T.. (2021). Cryostimulation for post-exercise recovery in athletes: a consensus and position paper. Front. Sports Act Living 3:688828. 10.3389/fspor.2021.688828 34901847 PMC8652002

[B5] Broatch J. R. Petersen A. Bishop D. J. (2017). Cold-water immersion following sprint interval training does not alter endurance signalling pathways or training adaptations in human skeletal muscle. Am. J. Physiol. 313, R372–R384. 10.1152/ajpregu.00434.2016 28679683

[B6] Broatch J. R. Poignard M. Hausswirth C. Bishop D. J. Bieuzen F. (2019). Whole-body cryotherapy does not augment adaptations to high-intensity interval training. Sci. Rep. 9:12013. 10.1038/s41598-019-48518-1 31427654 PMC6700067

[B7] Brook M. S. Wilkinson D. J. Mitchell W. K. Lund J. N. Szewczyk N. J. Greenhaff P. L.. (2015). Skeletal muscle hypertrophy adaptations predominate in the early stages of resistance exercise training, matching deuterium oxide-derived measures of muscle protein synthesis and mechanistic target of rapamycin complex 1 signaling. FASEB J. 29, 4485–4496. 10.1096/fj.15-273755 26169934

[B8] Cannon B. Nedergaard J. (2012). Yes, even human brown fat is on fire! J. Clin. Invest. 122, 486–489. 10.1172/JCI60941 22269320 PMC3266796

[B9] Clark D. R. Lambert M. I. Hunter A. M. (2012). Muscle activation in the loaded free barbell squat: a brief review. J. Strength Cond. Res. 26, 1169–1178. 10.1519/JSC.0b013e31822d533d 22373894

[B10] Coffey V. G. Hawley J. A. (2017). Concurrent exercise training: do opposites distract? J. Physiol. 595, 2883–2896. 10.1113/JP272270 27506998 PMC5407958

[B11] Cohen J. (1992). A power primer. Psychol. Bull. 112, 155–159. 10.1037/0033-2909.112.1.155 19565683

[B12] Collins S. M. Silberlicht M. Perzinski C. Smith S. P. Davidson P. W. (2014). The relationship between body composition and preseason performance tests of collegiate male lacrosse players. J. Strength Cond. Res. 28, 2673–2679. 10.1519/JSC.0000000000000454 24626136

[B13] Del Vecchio A. Casolo A. Negro F. Scorcelletti M. Bazzucchi I. Enoka R.. (2019). The increase in muscle force after 4 weeks of strength training is mediated by adaptations in motor unit recruitment and rate coding. J. Physiol. 597, 1873–1887. 10.1113/JP277250 30727028 PMC6441907

[B14] Earp J. E. Hatfield D. L. Sherman A. Lee E. C. Kraemer W. J. (2019). Cold-water immersion blunts and delays increases in circulating testosterone and cytokines post-resistance exercise. Eur. J. Appl. Physiol. 119, 1901–1907. 10.1007/s00421-019-04178-7 31222379

[B15] Esco M. R. Fedewa M. V. Cicone Z. S. Sinelnikov O. A. Sekulic D. Holmes C. J. (2018). Field-based performance tests are related to body fat percentage and fat-free mass, but not body mass index, in youth soccer players. Sports 6:105. 10.3390/sports6040105 30261675 PMC6316319

[B16] Fatourous I. G. Jamurtas A. Z. (2016). Insights into the molecular etiology of exercise-induced inflammation. J. Inflamm. Res. 9, 175–186. 10.2147/JIR.S114635 27799809 PMC5085309

[B17] Ferreira-Junior J. B. Bottaro M. Vieira A. Siqueira A. F. Vieira C. A. Durigan J. L.. (2014). One Session of partial-body cryotherapy (-110°C) improves muscle damage recovery. Scand. J. Med. Sci. Sports. 25, e524–530. 10.1111/sms.12353 25556301

[B18] Fonda B. Sarabon N. (2013). Effects of whole-body cryotherapy on recovery after hamstring damaging exercise: a crossover study. Scand. J. Med. Sci. Sports. 23, e270–e278. 10.1111/sms.12074 23614691

[B19] Fyfe J. J. Broatch J. R. Trewin A. J.. (2019). Cold water immersion attenuates anabolic signaling and skeletal muscle fiber hypertrophy, but not strength gain, following whole-body resistance training. J. Appl. Physiol. 127, 1403–1418. 10.1152/japplphysiol.00127.2019 31513450

[B20] Green D. J. Spence A. Rowley N. Thijssen D. H. Naylor L. H. (2012). Vascular adaptation in athletes: is there an 'athlete's artery'? Exp. Physiol. 97, 295–304. 10.1113/expphysiol.2011.058826 22179421

[B21] Haff G. G. Triplett N. T. (2016). Essentials of Strength Training and Conditioning, 4th Edn. Champaign, IL: Human Kinetics.

[B22] Halson S. L. Bartram J. West N. Stephens J. Argus C. K. Driller M. W.. (2014). Does hydrotherapy help or hinder adaptation to training in competitive cyclists? Med. Sci. Sports Exerc. 46, 1631–1639. 10.1249/MSS.0000000000000268 24504431

[B23] Haq A. Ribbans W. Baross A. (2021). The effects of age and body fat content on post downhill run recovery following whole body cryotherapy. Int. J. Environ. Res. Pub. Health 18:2906. 10.3390/ijerph18062906 33809147 PMC8001899

[B24] Hausswirth C. Louis J. Bieuzen F. Pournot H. Fournier J. Filliard J. R.. (2011). Effects of whole-body cryotherapy vs. far-infrared vs. passive modalities on recovery from exercise-induced muscle damage in highly-trained runners. PLoS ONE 6:e27749. 10.1371/journal.pone.0027749 22163272 PMC3233540

[B25] Hawley J. A. Lundby C. Cotter J. D. Burke L. M. (2018). Maximizing cellular adaptation to endurance exercise in skeletal muscle. Cell Metab. 27, 962–976. 10.1016/j.cmet.2018.04.014 29719234

[B26] Ihsan M. Abbiss C. R. Allan R. (2021). Adaptations to post-exercise cold water immersion: friend, foe, or futile? Front. Sports Act. Living 3:714148. 10.3389/fspor.2021.714148 34337408 PMC8322530

[B27] Ihsan M. Watson G. Choo H. C. Lewandowski P. Papazzo A. Cameron-Smith D.. (2014). Postexercise muscle cooling enhances gene expression of PGC-1α. Med. Sci. Sports Exerc. 46, 1900–1907. 10.1249/MSS.0000000000000308 24561815

[B28] Jaworska J. Laskowski R. Ziemann E. Zuczek K. Lombardi G. Antosiewicz J.. (2021). The specific judo training program combined with the whole body cryostimulation induced an increase of serum concentrations of growth factors and changes in amino acid profile in professional judokas. Front. Physiol. 9:627657. 10.3389/fphys.2021.627657 33633589 PMC7900507

[B29] Jaworska J. Micielska K. Kozłowska M. Wnorowski K. Skrobecki J. Radzimiński L.. (2018). A 2-week specific volleyball training supported by the whole body cryostimulation protocol induced an increase of growth factors and counteracted deterioration of physical performance. Front. Physiol. 9:1711. 10.3389/fphys.2018.01711 30555349 PMC6282029

[B30] Jaworska J. Rodziewicz-Flis E. Kortas J. Kozłowska M. Micielska K. Babińska A.. (2020). Short-term resistance training supported by whole-body cryostimulation induced a decrease in myostatin concentration and an increase in isokinetic muscle strength. Int. J. Environ. Res. Pub. Health. 17:5496. 10.3390/ijerph17155496 32751455 PMC7432449

[B31] Kraemer W. J. Duncan N. D. Volek J. S. (1998). Resistance training and elite athletes: adaptations and program considerations. J. Orth. Sports Phys. Ther. 28, 110–119. 10.2519/jospt.1998.28.2.110 9699161

[B32] Kraemer W. J. Looney D. P. (2012). Underlying mechanisms and physiology of muscular power. Strength Cond. J. 34, 13–19. 10.1519/SSC.0b013e318270616d

[B33] Lombardi G. Ziemann E. Banfi G. (2017). Whole-body cryotherapy in athletes: from therapy to stimulation. An updated review of the literature. Front. Physiol. 8:258. 10.3389/fphys.2017.00258 28512432 PMC5411446

[B34] Markovic G. Dizdar D. Jukic I. Cardinale M. (2004). Reliability and factorial validity of squat and countermovement jump tests. J. Strength Cond. Res. 18, 551–555. 10.1519/00124278-200408000-00028 15320660

[B35] Marrier B. Le Meur Y. Leduc C. Piscione J. Lacome M. Lgarza G.. (2018). Training periodization over an elite rugby sevens season: From theory to practice. Int. J. Sports. Physiol. Perf. 18, 1–9. 10.1123/ijspp.2017-0839 29952634

[B36] Mawhinney C. Low D. A. Jones H. Green D. J. Costello J. T. Gregson W. (2017). Cold-water mediates greater reductions in limb blood flow than whole body cryotherapy. Med. Sci. Sports Exerc. 49, 1252–1260. 10.1249/MSS.0000000000001223 28141620

[B37] McClenton L. S. Brown L. E. Coburn J. W. Kersey R. D. (2008). The effect of short-term vertimax vs. depth jump training on vertical jump performance. J. Strength Cond. Res. 22, 321–325. 10.1519/JSC.0b013e3181639f8f 18550943

[B38] Myer G. D. Kushner A. M. Brent J. L. Schoenfeld B. J. Hugentobler J. Lloyd R. S.. (2014). The back squat: a proposed assessment of functional deficits and technical factors that limit performance. Strength Cond. J. 36, 4–27. 10.1519/SSC.0000000000000103 25506270 PMC4262933

[B39] Partridge E. M. Cooke J. McKune A. Pyne D. B. (2019). Whole-body cryotherapy: potential to enhance athlete preparation for competition? Front. Physiol. 10:1007. 10.3389/fphys.2019.01007 31447697 PMC6691163

[B40] Petersen A. C. Fyfe J. J. (2021). Post-exercise cold water immersion effects on physiological adaptations to resistance training and the underlying mechanisms in skeletal muscle: a narrative review. Front. Sports Act. Living 3:660291. 10.3389/fspor.2021.660291 33898988 PMC8060572

[B41] Pilch W. Wyrostek J. Major P. Zuziak R. Piotrowska A. Czerwińska-Ledwig O.. (2020). The effect of whole-body cryostimulation on body composition and leukocyte expression of HSPA1A, HSPB1, and CRP in obese men. J. Cryobiol. 94, 100–106. 10.1016/j.cryobiol.2020.04.002 32289283

[B42] Roberts L. A. Raastad T. Markworth J. F. Figueiredo V. C. Egner I. M. Shield A.. (2015). Post-exercise cold water immersion attenuates acute anabolic signalling and long-term adaptations in muscle to strength training. J. Physiol. 593, 4285–4301. 10.1113/JP270570 26174323 PMC4594298

[B43] Schoenfeld B. J. (2010). The mechanisms of muscle hypertrophy and their application to resistance training. J. Strength Cond. Res. 24, 2857–2872. 10.1519/JSC.0b013e3181e840f3 20847704

[B44] Seitz L. B. Reyes A. Tran T. T. Saez de Villarreal E. Haff G. G. (2014). Increases in lower-body strength transfer positively to sprint performance: a systematic review with meta-analysis. Sports Med. 44, 1693–1702. 10.1007/s40279-014-0227-1 25059334

[B45] Stolen T. Chamari K. Castagna C. Wisløff U. (2005). Physiology of soccer: an update. Sports Med. 35, 501–536. 10.2165/00007256-200535060-00004 15974635

[B46] Turner A. (2011). The science and practice of periodization: a brief review. Strength Cond. J. 33, 34–46. 10.1519/SSC.0b013e3182079cdf31754845

[B47] Vecchio L. D. Daewoud H. Green S. (2018). The health and performance benefits of the squat, deadlift and bench press. MOJ Yoga Phys. Ther. 3, 40–47. 10.15406/mojypt.2018.03.00042

[B48] White G. E. Wells G. D. (2013). Cold-water immersion and other forms of cryotherapy: physiological changes potentially affecting recovery from high-intensity exercise. Extreme Physiol. Med. 2:26. 10.1186/2046-7648-2-26 24004719 PMC3766664

[B49] Wong P. L. Chaouachi A. Chamari K. Dellal A. Wisloff U. (2010). Effect of pre-season concurrent muscular strength and high-intensity interval training in professional soccer players. J. Strength Cond. Res. 24, 653–660. 10.1519/JSC.0b013e3181aa36a2 19816215

[B50] Yamane M. Teruya H. Nakano M. Ogai R. Ohnishi N. Kosaka M. (2006). Post-exercise leg and forearm flexor muscle cooling in humans attenuates eendurance and resistance training effects on muscle performance and on circulatory adaptation. Eur. J. Appl. Physiol. 96, 572–580. 10.1007/s00421-005-0095-3 16372177

[B51] Young W. B. Pryor J. F. Wilson G. J. (1995). Effect of instructions on characteristics of countermovement and drop jump performance. J. Strength Cond. Res. 9, 232–236. 10.1519/00124278-199511000-00005

[B52] Zembron-Lacny A. Morawin B. Wawrzyniak-Gramacka E. Gramacki J. Jarmuzek P. Kotlega D.. (2020). Multiple cryotherapy attenuates oxi-inflammatory response following skeletal muscle injury. Int. J. Environ. Res. Pub. Health 17:7855. 10.3390/ijerph17217855 33120891 PMC7663269

[B53] Ziemann E. Olek R. A. Kujach S. Grzywacz T. Antosiewicz J. Garsztka T. (2012). Five-day whole-body cryostimulation, blood inflammatory markers, and performance in high-ranking professional tennis players. J. Ath. Train. 47, 664–672. 10.4085/1062-6050-47.6.13 23182015 PMC3499891

